# Effects of a Thiosemicarbazide Camphene Derivative on *Trichophyton mentagrophytes*

**DOI:** 10.3390/molecules14051796

**Published:** 2009-05-13

**Authors:** Mirian Ueda Yamaguchi, Ana Paula Barbosa da Silva, Tânia Ueda-Nakamura, Benedito Prado Dias Filho, Cleuza Conceição da Silva, Celso Vataru Nakamura

**Affiliations:** 1 Programa de Pós-graduação em Ciências Farmacêuticas, Universidade Estadual de Maringá, PR, Brazil; E-mails: mirianuy@irapida.com.br (M-U.Y.), tunakamura@uem.br (T-U.N.), bpdfilho@uem.br (B-D.F.), cvnakamura@uem.br (C-V.N.); 2 Departamento de Química, Universidade Estadual de Maringá, PR, Brazil; E-mails: silvaapb@yahoo.com.br (A-B.S.), ccsilva@uem.br (C-C.S.)

**Keywords:** antifungal activity, thiosemicarbazide, camphene, *Trichophyton mentagrophytes*

## Abstract

Thiosemicarbazides are compounds known for their biological activity, particularly their antimicrobial properties, which include activity against fungi. The difficulty of treating fungal diseases induced us to assess the antifungal properties of some novel thiosemicarbazide compounds. We selected the natural products limonene and camphene as sources for the preparation of these new thiosemicarbazide derivatives. The compound *N*(4)-[2,2-dimethyl-3-methylnorbornane]-thiosemicarbazide (**TIO C**) showed an antifungal effect on *Trichophyton mentagrophytes*, with values of MIC = 55 μmol L^-1^ and MFC = 110 μmol L^-1^. Scanning-electron microscopy showed a decrease in mycelium development and morphological alterations of *T. mentagrophytes* cultured on nail fragments and treated with **TIO C**. In an attempt to discover its mode of action, we noted that ergosterol is apparently not a target of **TIO C** activity. An effect of **TIO C** on *T. mentagrophytes* cell walls and dividing cross-walls was shown by observed impairment of the fluorescence of tissues stained with calcofluor white, a specific marker for fungal chitin, suggesting that the compound can affect and damage the cell-wall structure or may interfere with its formation, during cell division, growth, and morphogenesis. This approach to the synthesis of new derivatives might provide interesting compounds with greater biological activity in pharmacological research.

## Introduction

Dermatophyte fungi are common pathogens that require keratin for growth, and invade keratinous tissues. Members of the genus *Trichophyton* are among the dermatophytes that are most often implicated in superficial cutaneous infections. The species *T. rubrum* and *T. mentagrophytes* are responsible for about 90% of chronic dermatophyte infections, showing a common pattern of association with tinea cruris, tinea pedis, tinea manuum, tinea barbae, and onychomycosis [1,2,3,4,5]. *T. rubrum* is the most prevalent dermatophyte species isolated from nails, followed by *T. mentagrophytes*; it is also frequently the etiological agent of tinea pedis (athlete’s foot) [4], and has demonstrated elevated resistance to commercial antifungal drugs [5,6]. The number of people affected by dermatophytes in the last three decades has increased; the reasons for this increase include the aging population, increased numbers of immunocompromised patients (transplants, HIV), diabetes, and other circumstances that affect the immunity of the general population [7,8,9].

Dermatomycosis is a nonfatal condition, but is difficult to eradicate, often necessitating long-term treatment. Although the antifungal agents ketoconazole, griseofulvin and, more recently, allylamines and triazoles have been used to treat dermatomycoses, resistance to the different drugs appears very frequently. This is mainly because different tineas (produced by diverse etiological agents) tend to be empirically treated with the same drugs [4] and as a consequence the dermatomycoses tend to persist, greatly diminishing the quality of life of persons suffering from these infections [10]. Since one of the strategies for avoiding the appearance of antifungal resistance is the treatment of fungal infection with the appropriate antifungal agent when the etiological agent is known, new antifungal agents that selectively inhibit a single fungal species are urgently needed, and will help to overcome the above problems [11].

In the search of powerful antimicrobial agents, the biological activity of thiosemicarbazide compounds has been investigated, and they have usually shown inhibitory activity against some species of bacteria, fungi, and protozoa [12]. The attention to thiosemicarbazide derivatives is justified because *in vitro* biological evaluation indicates that these derivatives have antitrypanosomal, antimalarial, and antiamoebic activity in non-cytotoxic concentrations to mammalian cells [13,14].

Compounds that are potentially useful due to their pharmacological properties have often been used as chiral starting materials for the enantioselective synthesis of biologically active derivatives. Among the huge diversity of chemical structures found in nature, the large monoterpene family has provided many examples of renewable natural products that meet the criteria of non-toxicity, high enantiopurity, and low cost required for new drug candidates. In this study, aiming at discovering structures endowed with antifungal activity, we selected *R-*(+)-limonene and (-)-camphene as sources for preparation of new thiosemicarbazides.

In recent years, several thiosemicarbazide derivatives have been synthesised and their biological activities have been explored [15], but few experimental data about their mechanisms of action have been reported. The present study sought to investigate the antifungal activity and to understand the inhibitory mechanism of a novel thiosemicarbazide derivative, the compound *N*(4)-[2,2-dimethyl-3- methylnorbornane]-thiosemicarbazide (**TIO C**), against *T. mentagrophytes.*

## Results and Discussion 

### Relationship between the antifungal activity and chemical structure of thiosemicarbazides

Thiosemicarbazide derivatives are reported to show biological activity, including antibacterial, antifungal, anti-HIV, analgesic, anti-inflammatory, and anti-tumour effects [16,17,18,19,20,21,22]. In turn, the natural products limonene and camphene have been reported to possess biological activity against Gram-positive and Gram-negative bacteria [23,24,25,26]. In view of these properties, our study envisaged that the combined effect of some of these compounds might result in increased activity.

The results of antifungal assays showed clear differences among the five compounds analysed (Table 1). The compounds camphene and limonene showed no effect on *T. mentagrophytes* even at the highest concentration tested, 735 μmol L^-1^. 

The MIC value of thiosemicarbazide without a substituent group (**TIO**) was 548 μmol L^-1^, whereas the MFC was greater than 735 μmol L^-1^. The limonene derivative, **TIO L**, showed moderate activity, with MIC and MFC values of 110 μmol L^-1^ and 220 μmol L^-1^, respectively. The camphene derivative (**TIO C**) showed the strongest effect on *T. mentagrophytes*, with values of MIC 55 μmol L^-1^ and MFC 110 μmol L^-1^. Fluconazole was used as the standard drug, and showed MIC 5 μmol L^-1^. The experiment was repeated three times for each compound. 

The chemical structural differences observed among the three thiosemicarbazides (**TIO**, **TIO C**, and **TIO L**) are attributed to the character of the substituent group: hydrogen for **TIO**, limonene for **TIO L**, and camphene for **TIO C** (chemical structures are shown in Table 1). Limonene and camphene belong to the same monoterpene class and are highly hydrophobic, and their incorporation into the thiosemicarbazide nucleus tends to significantly increase the hydrophobicity.

The remarkable antifungal features of **TIO C** and **TIO L** may be due to their more-hydrophobic character, and the absence of the substituent group for **TIO** explains the smaller antifungal effect. A previous report by Maccioni and co-workers [27,28] showed the same relationship between structure and activity in thiazole derivatives, where the stronger lipophilic character probably led to more-effective antifungal derivatives. Another study showed the relationship between the hydrophobic character of a small protein and its antifungal activity, reported by Peng *et al*. [29], who produced mutant genes of a specific seed to obtain a change in the protein-surface hydrophobicity, and showed that a hydrophobic surface is essential to increase the antifungal property.

### Effects on hyphae structure and inhibitory activity of TIO C on the invasion of nails

**Figure 1:**
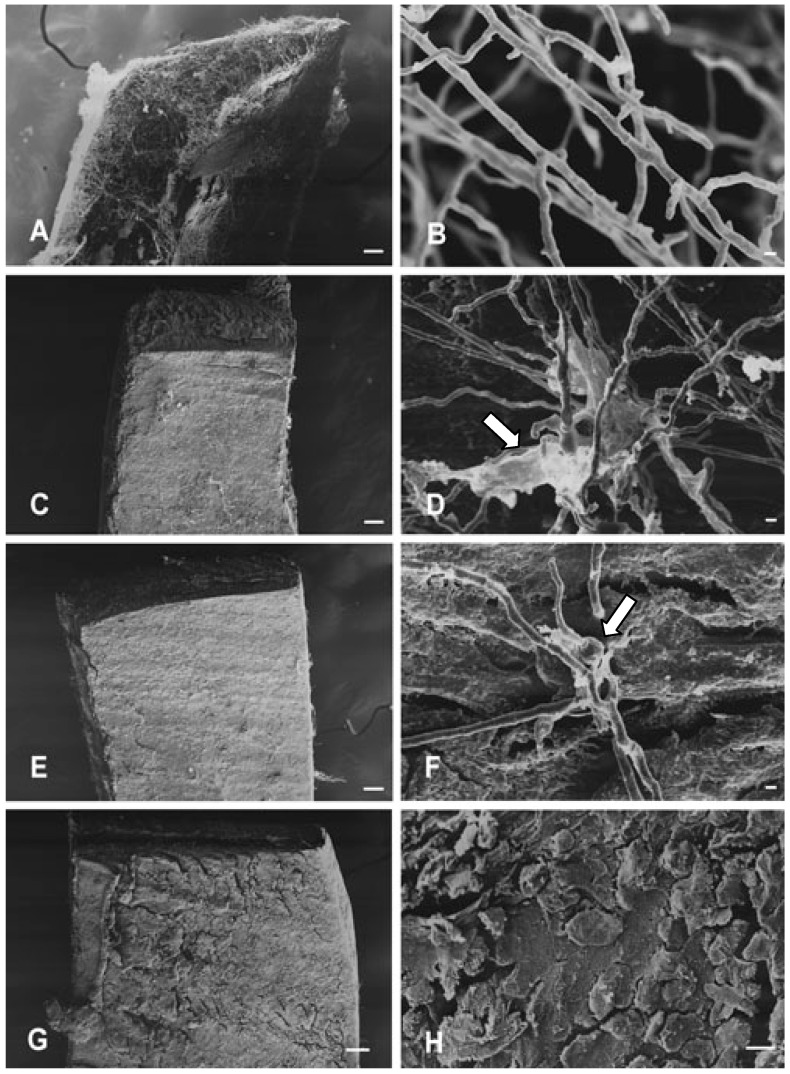
Scanning-electron microscopy showing mycelial structures of *T. mentagrophytes* cultured on nail fragments for 72 h, at 28 °C: (A-B) Control, (C-D) Treatment with **TIO C** 55 μmol L^-1^. (E-F) Treatment with **TIO C** 110 μmol L^-1^. (G-H) Treatment with **TIO C** 220 μmol L^-1^. View of nail fragment in (A) colonised by *T. mentagrophytes.* (C) and (E) show the inhibitory effect of TIO C on the invasion of nails. In (D) and (F) arrows indicate excretion of fibrillar materials and swollen structure. Figures (G) and (H) show that no mycelial growth can be seen on nail scales. Bars: 100 μm (A,C,E,G); 10 μm (H); 1 μm (B,D,F).

Fungicidal drugs are often preferred over fungistatic drugs for treatment of dermatophytic fungal infections, because fungi recur more often when fungistatic rather than fungicidal drugs are used. The most effective compound appraised in this study (**TIO C**) showed fungistatic action at low doses and fungicidal activity at high concentrations. These effects can be observed in Figure 1, which shows scanning-electron microscopy (SEM) images of *T. mentagrophytes* cultured on nail fragments after treatment with **TIO C**. 

Figure 1A and Figure 1B show untreated controls, with abundant growth and normal hyphae structures. The inhibitory effect of **TIO C** at 55 μmol L^-1^ and 110 μmol L^-1^ concentrations is shown in Figure 1(C,D) and Figure 1(E,F), respectively. In Figure 1D and Figure 1F, arrows indicate excretions of fibrillar materials, and swollen hyphae. The presence of hyphae with swollen portions may reflect a weakening of the cell wall. Microscopy images (Figures 1G, H) show that no mycelia growth was observed on nail scales at 220 μmol L^-1^. The results for growth inhibition and morphological alterations were observed in two independent experiments. 

### Absence of linkage between ergosterol and TIO C

**Figure 2:**
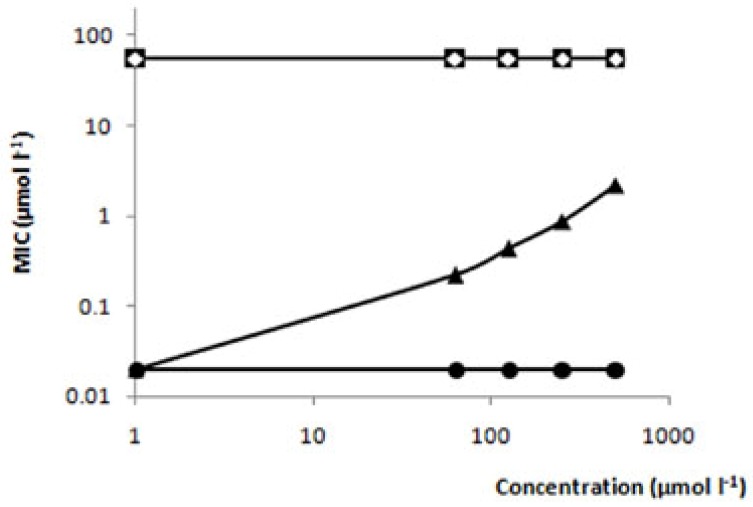
Effect of ergosterol on the MIC of **TIO C** against *T. mentagrophytes.* (■) **TIO C**; (◇) **TIO C** + Ergosterol; (●) Amphotericin B; (▲) Amphotericin B + Ergosterol.

The mode of action of **TIO C** is unknown. Amphotericin B is a well-known membrane-disruptive agent, which has a direct effect on membranes and is lethal to fungi. It forms stable pores in the membrane by complexing with ergosterol, which results in permeability to several ions [30]. In order to test the possibility that **TIO C** might act in a similar manner to amphotericin B, *T. mentagrophytes* fungus was treated with **TIO C**, and cultured in the presence and absence of exogenous ergosterol [31]. The results demonstrated that in this assay, the same value of MIC (55 µmol L^-1^) was maintained with (Figure 2■), and without ergosterol (Figure 2◇), showing that **TIO C** does not complex with ergosterol, and suggesting that its mode of action is probably by another mechanism or on another target. On the other hand, amphotericin B in the presence of exogenic ergosterol (Figure 2▲) showed MIC enhancement, confirming the mode of action of amphotericin B, by binding to membrane ergosterol. This experiment was carried out in duplicate, in three independent experiments.

### Effects of TIO C on cell wall structure 

**Figure 3:**
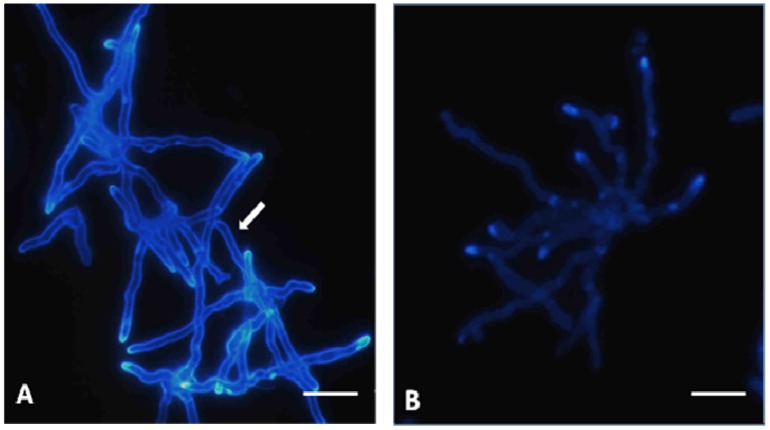
Immunofluorescence microscopy showing hyphae of *T. mentagrophytes* stained by calcofluor white: (A) Control, arrows indicate intense labelling of the cell wall by calcofluor white; (B) Hyphae after treatment with **TIO C** 55 μmol L^-1^ show weak labelling of the cell wall. Bars: 10 μm.

The fungal cell wall is a complex structure that is often composed of chitin, 1,3-β- and 1,6-β-glucan, mannan, proteins, and other polymers, although the wall composition varies markedly among species of fungi [18,32]. The derivative in this study, **TIO C**, showed different activities on other species of fungi (data not shown). **TIO C** does not show fungicidal activity against *Candida albicans* ATCC 10231 or *Microsporum gypseum* ATCC 14683. The activity of **TIO C** on the *T. mentagrophytes* cell wall could be observed by staining with calcofluor white, a marker of fungal chitin. The control, cultured without **TIO C**, exhibited a brighter fluorescence of the fungus cell wall and dividing cross-walls (Fig. 3-A). In contrast, the cell wall of the fungus that had been cultured in the presence of **TIO C** at 55 μmol L^-1^ was not detected when stained with calcofluor (Figure 3-B). This suggests that **TIO C** can affect and damage the cell-wall structure or may interfere with its formation, during cell division, growth, and morphogenesis. This possibility merits further investigation.

## Experimental 

### General 

All melting points were determined using a Microquímica model MQAPF-301 apparatus, and are uncorrected. IR spectra were obtained using KBr pellets in an FT-IR BOMEM spectrophotometer. Low-resolution mass spectra were recorded by means of a Shimadzu-GC/MS model QP 2000A spectrometer at 70 eV equipped with a solids probe. The optical rotations were determined, in a CHCl_3_ solution, with a Perkin-Elmer Model 343 polarimeter at 25 °C. Proton nuclear magnetic resonance (^1^H-NMR) spectra were recorded using CDCl_3_ as a solvent, at ambient temperature, on a Varian Mercury (300 MHz) instrument with TMS as an internal standard. The chemical shifts (δ) are given in parts per million relative to TMS. Carbon-13 nuclear magnetic resonance (^13^C-NMR) spectra were recorded at 75 MHz with the same internal standard.

### Synthesis of thiosemicarbazides

**Scheme 1:**
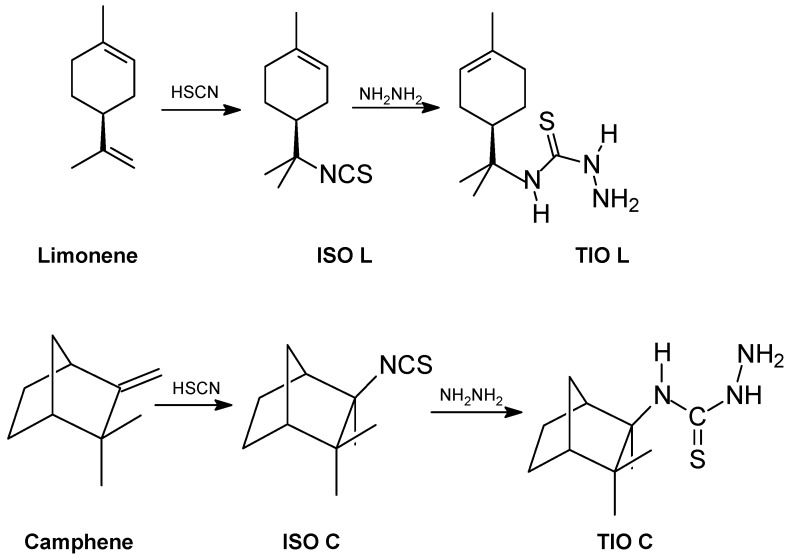
Synthetic routes for the thiosemicarbazides **TIO L** and **TIO C**.

The isothiocyanates, 2,2-dimethyl-3-(isothiocyanomethyl) norbornane isothiocyanate (**ISO L**) and *R*-1-methyl-4-(1-isothiocyano-4-isopropyl)-cyclohexene isothiocyanate (**ISO C**) were prepared according to the method of Silva [33], as shown in Scheme 1. A solution of hydrazine (5.15 g, 0.0491 mol) was dissolved in ethanol (25 mL), and then the isothiocyanomonoterpene (9.57 g, 0.0491 mol) was added. The mixture was stirred for 5 h at 90 °C, extracted with CHCl_3_, and washed with hexane.

*N(4)-[R-(1-Methyl-4-isopropylcyclohexene]-thiosemicarbazide* (**TIO L**): White crystals; yield 85%; mp. 125 -127 °C; [α]_D_ +24; IR (KBr): (NH) 3370, (C=S) 1504; EI-MS *m/z* 227 (M^+•^); ^1^H-NMR: δ 5.36 (1H, brs, H-2), 1.83-1.98 (2H, m, H-3), 2.56 (1H, m, H-4), 1.75-1.79 (2H, m, H-5), 1.92-2.05 (2H, m, H-6), 1.50 (3H, s, H-8), 1.48 (3H, s, H-9), 1.64 (3H, s, H-10); ^13^C-NMR: δ 134.3 (C-1), 120.7 (C-2), 26.7 (C-3), 41.4 (C-4), 24.3 (C-5), 30.8 (C-6), 58.2 (C-7), 24.2 (C-8), 24.6 (C-9), 23.5 (C-10) and 180.7 (C-3’) (C=S).

*N(4)-[2,2-Dimethyl-3-methylnorbornane]- thiosemicarbazide* (**TIO C**): White crystals; yield 85%; mp. 106 -109 °C; [α]_D_ -105; IR (KBr): (NH) 3261, (C=S) 1532; EI-MS *m/z* 227 (M^+•^); ^1^H-NMR: δ 5.36 (1H, brs, H-2 ), 1.83-1.98 (2H, m, H-3), 2.56 (1H, m, H-4), 1.75-1.79 (2H, m, H-5), 1.92-2.05 (2H, m, H-6), 1.50 (3H, s, H-8), 1.48 (3H, s, H-9), 1.64 (3H, s, H-10); ^13^C-NMR: δ 134.3 (C-1), 120.7 (C-2), 26.7 (C-3), 41.4 (C-4), 24.3 (C-5), 30.8 (C-6), 58.2 (C-7), 24.2 (C-8), 24.6 (C-9), 23.5 (C-10) and 180.7 (C-3’) (C=S).

### Strain and culture conditions

The *Trichophyton mentagrophytes* ATCC 11481 strain used in this study was obtained from the Instituto Oswaldo Cruz (FIOCRUZ, Rio de Janeiro, Brazil), and was subcultured in tubes containing sloping Dextrose Sabouraud Agar medium (Difco & BBL Co., Le Pont de Claix, France), for 14 days, at 28 °C. Sterile 0.85% saline was added to the tubes, agitated, and filtered, and the conidial solution was standardised at 10^4^ cells mL^-1^, with a haemacytometer.

### Fungal Inhibitory activity

Minimal inhibitory concentrations (MICs) were determined by at least three experiments performed in duplicate, using the broth micro-dilution procedure, according to the recommendations of the Clinical and Laboratory Standards Institute (CLSI, 2002) [34]. The test compounds were dissolved in dimethyl sulfoxide (DMSO), and further dilutions were prepared in 0.85% sterile saline. In RPMI 1640 medium (Sigma-Aldrich Chemical Co., Missouri, USA, 8.6 g L^-1^), after 72 h incubation, at 28 °C, the minimum inhibitory concentration (the lowest concentration of the compound that inhibited visible growth of fungus) was determined.

### Fungicidal activity

The minimal fungicidal concentrations (MFCs) of the compounds were obtained by a subculture of 10 μL from minimum-inhibitory-concentration wells, without visible growth, placed in Petri dishes containing Dextrose Sabouraud Agar medium. After incubation for 72 h at 28 °C, the minimum fungicidal concentration was determined by the lowest concentration without fungus growth.

### Scanning electron microscopy (SEM)

Cells were incubated in RPMI 1640 medium for 72 h at 28 °C with **TIO C** compound, in four tubes, at 55 μmol L^-1^,110 μmol L^-1^, 220 μmol L^-1^, and a negative control. The tubes were centrifuged, and the pellets washed twice with PBS (pH 7.2). The pellets were fixed with 2.5% glutaraldehyde and placed on cover slips (3 x 3 mm^2^) treated with poly-L-lysine. The cover slips were washed three times in 0.1 mol L^-1^ sodium cacodylate buffer and dehydrated in a graded ethanol series, critical-point dried, sputter-coated with gold, and assessed by means of a scanning electron microscope (Shimadzu SS 550).

### Nail fragments invasion test

The procedure described by Rashid *et al*. [35] was performed, with modifications. Healthy volunteers provided broken-off nails for this procedure. The nails were cut in pieces of about 3 x 3 mm^2^ and sterilised in an autoclave at 121 °C for 15 min. The sterilised nail fragments were pre-exposed to a suspension containing 10^5^ spores/mL of *T. mentagrophytes* for 1 h, at 28 °C. The nail fragments were then removed from the spore suspension and placed in a 24-well plate containing 500 μL of ion solution. **TIO C** solution was added to the wells at concentrations of 55 μmol L^-1^, 110 μmol L^-1^, and 220 μmol L^-1^. The plate was incubated for 72 h at 28 °C. The nail fragments were fixed with 2.5% glutaraldehyde dehydrated in a graded ethanol series, critical-point dried, sputter-coated with gold, and examined by means of a scanning electron microscope (Shimadzu SS 550).

### Fungus cell wall integrity analysis

Calcofluor White stains the cell wall of fungi, binding to chitin chains. Calcofluor White (Sigma Biochemical, St. Louis, MO, USA) was utilised to stain the cell walls of *T. mentagrophytes*, in order to show the effect of TIO C by means of the fluorescence of the stain. Solutions with MIC and sub-MIC of TIO C were placed in 24-well plates containing coverslips and 500 μL of RPMI medium. A suspension with inoculum of 2,000-3,000 spores was added to each well, and incubated at 28 °C for 48 h. Spores not treated with **TIO C** or treated with the solvent DMSO were included as controls. The medium was drained off the wells, and 250 µL of 0.1 mmol L^-1^ Calcofluor White solution was added to stain the cell walls. After 15 min of incubation at room temperature, the coverslips were rinsed with 0.1 mol L^-1^ phosphate-buffered saline (PBS, pH 7.2). The coverslips were observed in a fluorescence microscope (Zeiss Axiocam – Axioshop 2 plus).

### Ergosterol effect assay

The MIC of **TIO C** against *T. mentagrophytes* was determined as described previously [31], in the presence and in the absence of ergosterol (Sigma Chemical, St Louis, MO, USA), at the concentrations 60, 125, 250, and 500 µmol mL^-1^, added to RPMI 1640 medium. Amphotericin B (Cristalia Ltda, São Paulo, Brazil) was used as the control drug. At least three experiments were performed in duplicate.

## Conclusions

The thiosemicarbazide derived from camphene (**TIO C**) shows a greater antifungal effect when compared with thiosemicarbazide without a substituent group (**TIO**). The incorporation of camphene into the thiosemicarbazide molecule is responsible for the increase in antifungal activity. **TIO C** apparently acts on the fungus cell wall, as substantiated by calcofluor assay and SEM images showing distorted and swollen hyphae, suggesting a weakening of the cell wall. However, further investigations are necessary to elucidate the mode of action of **TIO C**, seeking to make use of this derivative in combating fungal diseases, especially those caused by the fungus *T. mentagrophytes*, which are difficult to treat and are increasing in prevalence worldwide.

## Figures and Tables

**Table 1:** Chemical structure and Minimal Inhibitory Concentrations (MIC) and Minimal Fungicidal Concentrations (MFC) of camphene, limonene, and the thiosemicarbazides TIO, TIO C, and TIO L against *T. mentagrophytes.*

Compound	Structure	MIC (μmol L^-1^)	MFC (μmol L^-1^)
Limonene	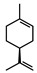	>735	>735
Camphene	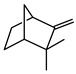	>735	>735
TIO	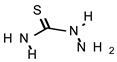	548	>735
TIO L	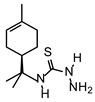	110	220
TIO C	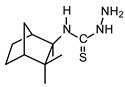	55	110
